# A PERSON-SPECIFIC APPROACH TO EMOTION REGULATION FLEXIBILITY ACROSS ADULTHOOD

**DOI:** 10.1093/geroni/igae098.1481

**Published:** 2024-12-31

**Authors:** Tammy English, Tabea Springstein

**Affiliations:** Washington University in St. Louis, St. Louis, Missouri, United States; Washington University in St. Louis, St. Louis, Missouri, United States

## Abstract

Early work on emotion regulation typically focused on how often or how much individuals use “adaptive” strategies (e.g., cognitive reappraisal) or “maladaptive” strategies (e.g., expressive suppression”), largely finding little evidence of age differences in strategy use. However, there is growing attention to the importance of flexibly adjusting regulation efforts based on constraints and affordances of the situation, a skill that may increase with life experience. In the present research, we build on these recent theoretical innovations by considering both the “frequency perspective” and “flexibility perspective” in an adult lifespan sample. To do so, we utilize a novel statistical approach that models emotion regulation strategy use and person-specific strategy-situation fit associations with well-being. In an experience sampling study, 290 adults aged 25 to 85 years were prompted 6x/day for 10 days to report on their emotion regulation strategy use and various aspects of their current situation. Bayesian mixed-effect location scale modeling was used to simultaneously assess the relationships of flexibility and frequency of emotion regulation strategy use. Results suggest that individuals with higher well-being used some strategies flexibly and more frequently, some strategies flexibly but not more or less frequently, and some strategies more frequently but not flexibly. This work highlights the value of considering person-specific approaches to emotion and the need for attention to both flexibility and overall adaptiveness of regulation strategies. Work on adult development in emotion could benefit from incorporating idiographic perspectives that take individual differences seriously, rather than operating from more of a one-size-fits-all view of aging.

